# Differentiation of sphingomyelin and cholesterol by hyperspectral mid-infrared detection of single-bond vibrational modes in the fingerprint region

**DOI:** 10.1038/s41592-026-03025-w

**Published:** 2026-03-30

**Authors:** Francesca Gasparin, Alexander Prebeck, Alice Soldà, Nasire Uluç, Sarah Glasl, Constantin Berger, Miguel A. Pleitez, Vasilis Ntziachristos

**Affiliations:** 1 Chair of Biological Imaging, Central Institute for Translational Cancer Research (TranslaTUM), School of Medicine and Health & School of Computation, Information and Technology, Technical University of Munich, Munich, Germany; 2 Institute of Biological and Medical Imaging, Bioengineering Center, Helmholtz Zentrum München, Neuherberg, Germany; 3 Department of Physics, Middle East Technical University, Ankara, Turkey; 4 Institute of Computational Biology, Bioengineering Center, Helmholtz Zentrum München, Neuherberg, Germany; 5 DZHK (German Centre for Cardiovascular Research), partner site Munich Heart Alliance, Munich, Germany

**Keywords:** Cellular imaging, Imaging

## Abstract

Lipids play a central role in a multitude of biological functions associated with cancer, obesity, diabetes, cardiovascular and neurological pathologies. However, sensing and mapping of lipid classes in living cells remains challenging. Here we introduce a label-free approach to lipid imaging, which differentiates lipid species in living cells by hyperspectral mid-infrared detection of single-bond vibrational modes within the fingerprint region. Hyperspectral fingerprint optoacoustic microscopy is shown to resolve phosphatidylcholine, sphingomyelin or cholesterol in test samples and in synthetic giant unilamellar vesicles used as models of cell membranes. Then, mapping of total cholesterol and sphingomyelin content and accumulation dynamics are demonstrated in living cells. Hyperspectral fingerprint optoacoustic microscopy demonstrates sensitivity not only in discerning lipids with substantially different chemical structures, such as cholesterol and phospholipids, but also lipids that are chemically similar, such as sphingomyelins and glycerophospholipids.

## Abstract (web summary)

Hyperspectral fingerprint optoacoustic microscopy enables the differentiation of different lipid classes in living cells, which is of interest in the study of lipid metabolism in health and disease.

## Main

Besides being used as energy storage and structural cell membrane elements, lipids are implicated in multiple pathways of tissue function or disease progression. With an increasing understanding of the diversity of these roles, there is growing research to elucidate the implications of lipids in cancer, obesity, diabetes, neurological or cardiovascular diseases. Lipidomics and genome-wide association studies have revealed diverse disease-associated defects in lipid pathways; however, the need for observations of specific lipid species in living systems is necessary to better decipher dynamic relations between lipid metabolism, disease evolution and the effect of environmental and lifestyle influences^1^.

Optical microscopy methods, such as photo-activated localization microscopy, has used plasmid-encoded fluorescent toxins and proteins for targeting cholesterol (Chol) and sphingomyelin (SM)-enriched microdomains^2,3^. Fluorescence resonance energy transfer, stochastic optical reconstruction microscopy^4^ and stimulated emission depletion microscopy^5^ have also shown spatiotemporal observations of lipid species. Nevertheless, lipid staining may be limited by the efficiency of delivering a label into the living cells, the selectivity and specificity of the label to specific lipids or lipid classes, the ability to multiplex concurrent targeting of multiple lipid species and the possible alterations of cellular function by the introduction of the label^6–8^. Moreover, development of labels requires laborious validation and may nevertheless lead to insufficient performance, for example, in labeling Chol in living cells^8^.

These challenges have steered attention to label-free techniques for lipid sensing. Third harmonic generation^9^ and optical^10^ or optoacoustic methods^11–18^ in the 900–1,800 nm spectral region can sense lipids without the use of labels but do not separate lipid classes. Mid-infrared (mid-IR) spectroscopy, at the 2,900–2,800 cm^−1^ range, also reveals C–H single-bond stretching modes representative of lipids^19^ but was not found sufficient to differentiate lipid species. Label-free detection of intracellular unsaturated and saturated lipid droplets has been proven possible with stimulated Raman scattering microscopy in the high wavenumber (3,050−2,800 cm^−1^) and fingerprint region (1,780−1,200 cm^−1^) in bacteria and cancer cells^20,21^. Coherent anti-Stokes Raman scattering (CARS) improves Raman detection sensitivity of lipid droplets in living adipocytes^22^. However, applied to the single-bond C–H stretching band (3,100–2,800 cm^−1^), common in a multitude of molecules including carbohydrates, nucleic acids and the hydrocarbon chain of the majority of lipids, CARS attains reduced specificity to different lipid classes owing to signal crosstalk^23^. Integration of hyperspectral stimulated raman scattering with *k*-means clustering and multivariate curve resolution has been proposed to improve the specificity but is also challenged by low sensitivity^24^. Indeed, so far, in vivo detection of different lipid classes, such as Chol, has only been possible when hyperspectral stimulated raman scattering was combined with isotopic labeling or applying computational unmixing methods to resolve the spectral overlap in the C–H stretching region^25,26^.

In the quest for label-free sensing of lipid species in living cells, we investigated optoacoustic detection in the mid-IR fingerprint region. Fingerprint bands reveal fine biomolecular content but exhibit weak Raman scattering cross-sections^25^. Conversely, using photon absorption rather than scattering, mid-IR cross-sections are stronger than Raman^19^, potentially leading to higher sensitivity and signal-to-noise ratio. To perform this investigation, we introduce hyperspectral fingerprint optoacoustic microscopy (hyFOPM), operating at 1,730–900 cm^−1^, aimed at sensing the specific single-bond vibrational modes of lipids. The system also allows scanning of the single-bond C–H stretching region (2,932−2,770 cm^−1^), for reference purposes.

Here, we show hyFOPM specificity to different lipid species with controlled phantoms and with three-dimensional (3D) synthetic giant unilamellar vesicles (GUVs), the latter serving as models for the cell membrane. In particular, we unmixed lipid contributions in GUVs composed of different mixes of Chol, SM and unsaturated phosphatidylcholine (1,2-dioleoyl-*sn*-glycero-3-phosphocholine, DOPC). Chol and SM were selected because they play a major role in several cellular functions and have a different chemical structure, whereby DOPC was selected owing to its chemical structural similarity to SM, as a control molecule. Results were contrasted to attenuated total reflectance–Fourier transform infrared (ATR–FTIR) measurements. Then, applied to live cell imaging, hyFOPM followed and separated the dynamics of SM and Chol in human embryonic kidney (HEK) cells and lung adenocarcinoma cells. We discuss the implications of label-free separation of lipids in the study of cancer, neurodegenerative, cardiovascular and other diseases and the opportunities that hyFOPM detection opens in the study of metabolic processes^27^.

## Results

### Lipid phantoms

**Fig. 1:**
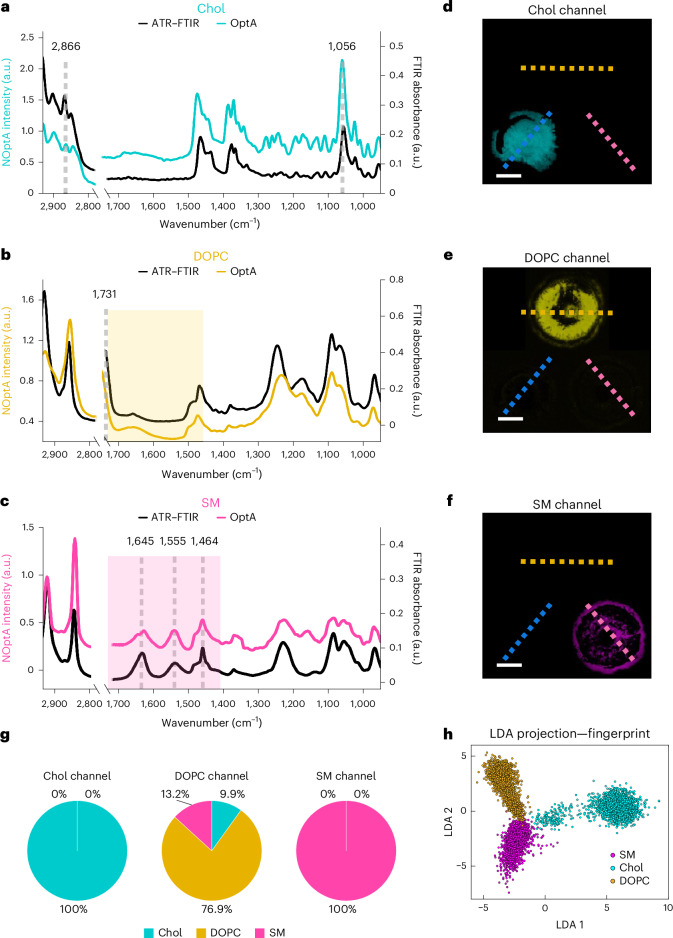
Imaging and spectroscopy of a 2D lipid phantom by hyFOPM. **a**–**c**, Comparison of mid-IR absorption hyperspectra of dried Chol (**a**), DOPC (**b**) and SM [SM 18:0] (**c**) measured by hyFOPM and by ATR–FTIR spectroscopy. Yellow and pink shading highlights significant spectral differences between DOPC and Chol. **d**–**f**, Contrast separation between Chol (**d**), DOPC (**e**) and SM (**f**) resulting from linear unmixing of the 2D phantom spectral images in the fingerprint region. **g**, Pie charts illustrating the crosstalk between unmixed images in **d** to **f**, presented as the mean value of the line profile drawn in **d** to **f**. Chol, DOPC and SM exhibited crosstalk levels of ~0%, 23% and 0%, respectively. **h**, LDA projection of the hyFOPM pixels acquired in the fingerprint region. Scale bar, 1 mm. *N* = 3. NOptA, normalized optoacoustic. Source data

**Extended Data Fig. 1:**
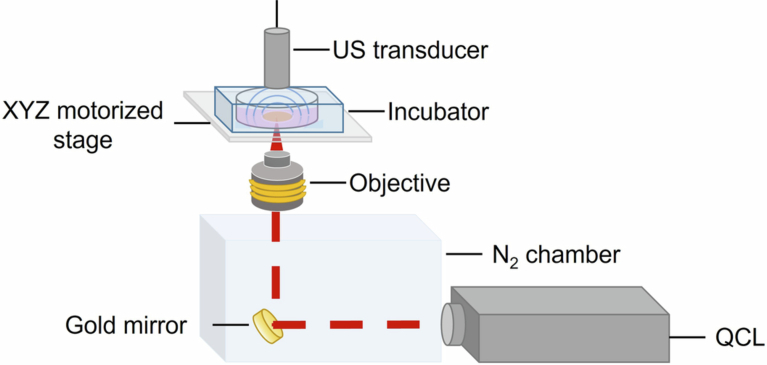
A schematic overview of the hyperspectral fingerprint optoacoustic microscopy (hyFOPM) system. The hyFOPM setup employs a tunable quantum cascade laser (QCL), operating over the spectral range of 3.4–11.0 μm, to provide mid-infrared excitation suitable for hyperspectral imaging. Optoacoustic signal detection is achieved using a piezoelectric focused ultrasound (US) transducer with a central frequency of 25 MHz.

**Extended Data Fig. 2:**
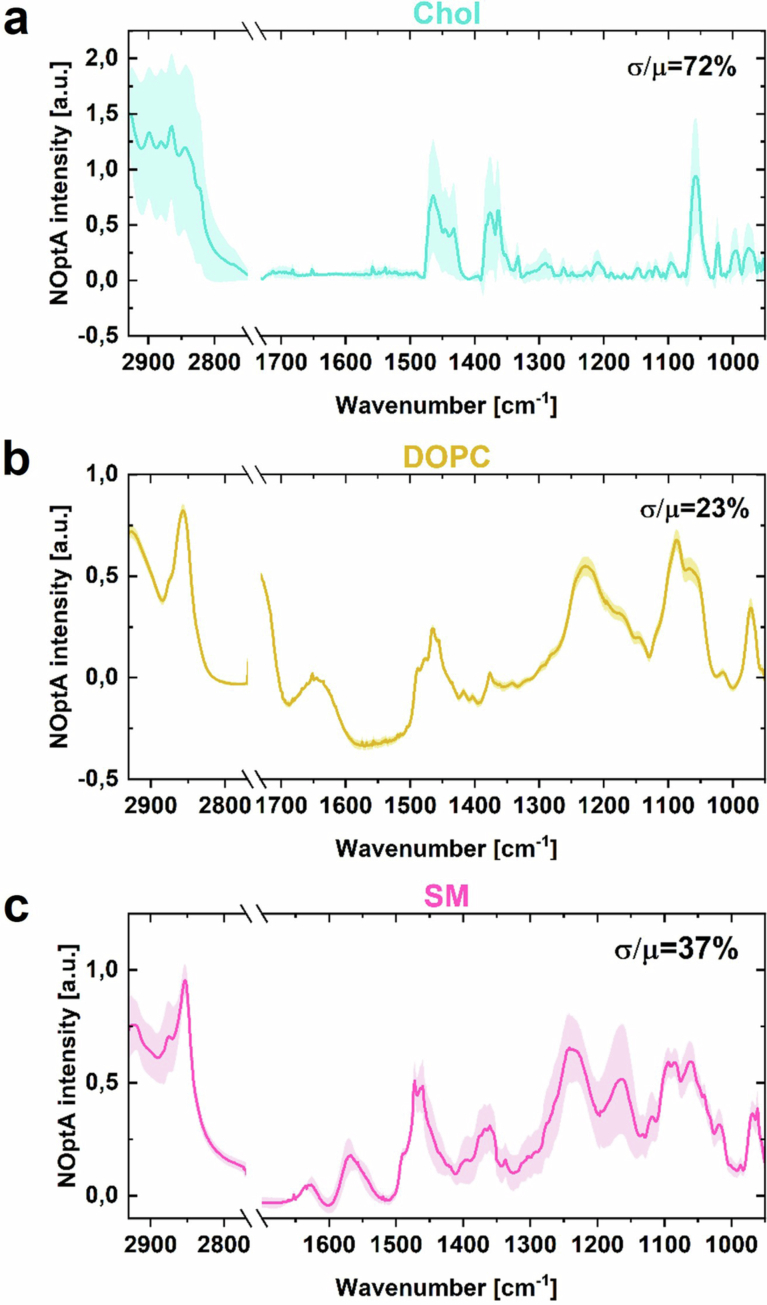
Bi-dimensional lipid phantom spectral reproducibility. Mean (μ) and standard deviation (σ) of spectra collected at different spatial location within the wells of the bi-dimensional lipid phantom. The coefficient of variation is expressed as σ/μ. **a)** Mean and standard deviation of four cholesterol (Chol) spectra. **b)** Mean and standard deviation of ten 1,2-dioleoyl-sn-glycero-3-phosphocholine (DOPC) spectra. **c)** Mean and standard deviation of ten sphingomyelin (SM) spectra. NOptA: Normalized Optoacoustic.

HyFOPM was developed (Extended Data Fig. 1) and applied to measuring a two-dimensional (2D) lipid phantom (Fig. 1a–c), composed of a four-well carbon tape grid, each well containing Chol, DOPC, SM and water (Fig. 1d–f and Supplementary Video 1). HyFOPM spectra were acquired over the 1,730–900 cm^−1^ (fingerprint) and 2,932−2,770 cm^−1^ bands, with 2 cm^−1^ spectral resolution over 7 min, and showed good correspondence with ATR–FTIR spectra acquired from the same samples (Fig. 1a–c) and to mid-IR vibrational assignments from literature^28–31^. The Chol chemical structure with its four sterol rings differs substantially from SM, and these dissimilarities are reflected on the spectral signatures resolved, for example, the strong 1,056 cm^−1^ peak ascribed to the deformation mode of the sterol rings structure^29^ (Fig. 1a). Conversely, both DOPC and SM contain fatty acid chains and a polar head group with phosphate and choline, yielding similar spectra in the single-bond C–H stretching region but different spectral features in the fingerprint region. DOPC uniquely shows a peak at 1,731 cm^−1^ typical of the C=O stretching mode of ester groups (Fig. 1b), while SM is characterized by three peaks (at 1,645, 1,555 and 1,464 cm^−1^), assigned to the amide bond vibrations (amide I and II), that are absent from the DOPC spectrum, and to the fatty acid CH_2_ bending (Fig. 1c). These spectral differences in the fingerprint region between SM and DOPC were validated by reproducibility studies (Extended Data Fig. 2).

To examine the correspondence of the spectra collected to the mid-IR vibrational assignments in literature, we analyzed hyFOPM spectra taken from the 2D phantoms at 22 wavenumbers (Table 1) spanning the C–H stretching and the fingerprint spectral region (Supplementary Video 1). The wavenumbers were selected to indicate key features (for example, peaks) seen on the SM, Chol and DOPC spectra in Fig. 1a–c.

**Table 1:** Mid-IR optoacoustic spectral bands with their relative mid-IR vibrational assignments

Frequencies (wavenumber in cm^−1^)	Type of vibrations and assignment	Comment
2,932	*ν*_as_ CH_2_, *ν*_as_ CH_3_	Chol
2,923	*ν*_as_ CH_2_ of acyl chain	DOPC
2,916	*ν*_as_ CH_2_	SM
2,900	*ν*_s_ CH_2_	Chol
2,866	*ν*_s_ CH_2_	Chol
2,852	*ν*_s_ CH_2_ of acyl chain	DOPC−SM
2,848	*ν*_s_ CH_2_ and CH_3_	Chol
1,731	*ν* C=O ester group	DOPC
1,645	C=O amide I	SM
1,555	*δ* N–H, *ν* C–N amide II	SM
1,464	*α* CH_2_ acyl chain	All
1,375	*β*_s_ CH_3_	All
1,327	*π* CH_2_	Chol
1,234	*ν*_as_ PO_2_^−^	DOPC–SM
1,174	*ν* C–O, *ν*_s_ C–C–O	DOPC
1,164	*ν* C–O, *ν*_s_ C–C–O	SM
1,087	*ν*_s_ PO_2_^−^	DOPC–SM
1,064	*ν*_as_ C–C–O, *ν* C–O–H	DOPC–SM
1,056	*δ* C–H ring	Chol
1,023	β C–Hin plane	Chol
964	*ν*_as_ N^+^(CH_3_)_3_, β C=C	DOPC–SM
958	β =C–H	Chol

Data are from ref. ^29^. α, scissoring; β, bending; δ, deformation; ν, stretching; π, wagging; ρ, rocking; τ, twisting (s, symmetric; as, asymmetric).

**Extended Data Fig. 3:**
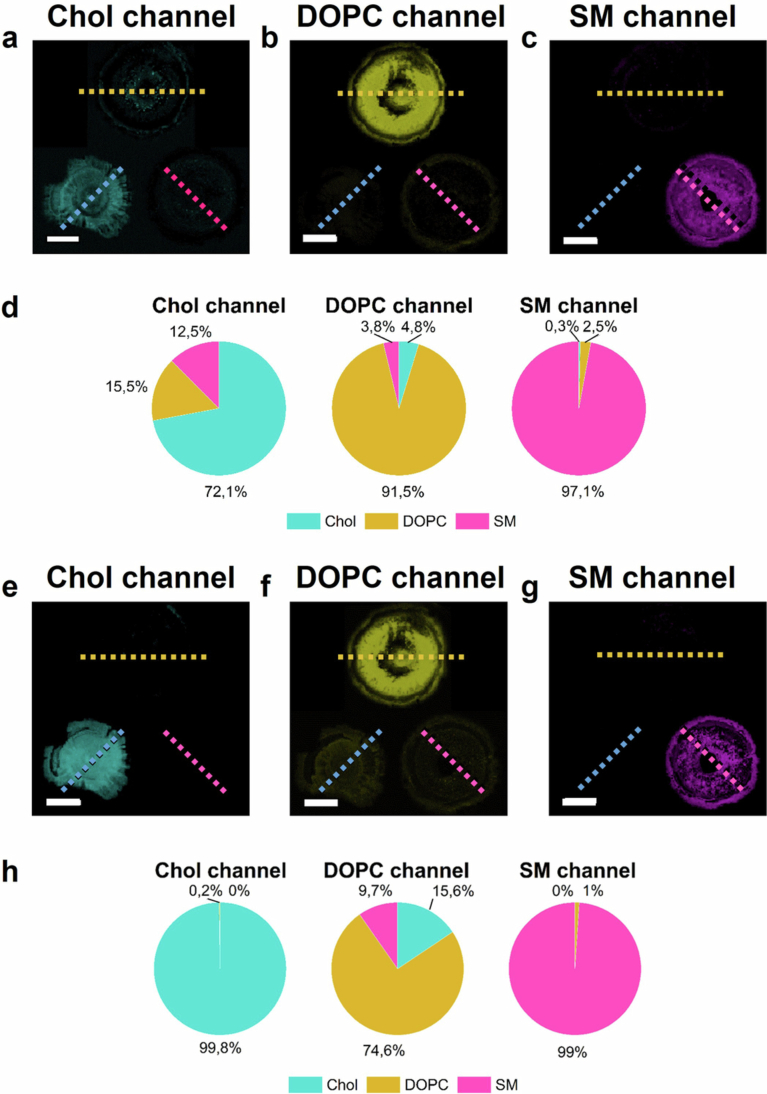
Linear unmixing and crosstalk pie charts. **a-c)** Lipids’ maps resulted from linear unmixing of hyFOPM images acquired in the CH-stretching region (cholesterol (Chol) in (**a**), phosphatidylcholine (DOPC) in (**b**), SM (SM) in (**c**)). **d)** Pie charts representing the cross talk of the three lipid channels in **a-c**. Chol, DOPC, SM exhibited crosstalk levels of ~28%, 9%, and 3%, respectively. **e-g)** Lipids’ maps resulted from linear unmixing of hyFOPM images acquired in both the CH stretching and fingerprint region (Chol in (**e**), DOPC in (**f**) and SM in (**g**)). **h)** Pie charts representing the cross talk of the three lipid channels in **e-g**. Chol, DOPC, SM exhibited crosstalk levels of ~1%, 25%, and 1%, respectively. Scale bar: 1 mm, N = 3.

In a next step, we aimed to investigate the identification and separation of the phantom lipids, water and carbon tape contributions from each other. Linear unmixing was performed at (1) the 22 wavenumbers in Table 1, (2) only 7 wavenumbers in the C–H stretching region and (3) only 15 wavenumbers in the fingerprint spectral region. In Fig. 1d–f, we show the unmixed hyFOPM images at the (3) fingerprint region, whereby Chol is highlighted in cyan (Fig. 1d), DOPC in yellow (Fig. 1e) and SM in magenta (Fig. 1f). For comparison, we report the results of linear unmixing applied respectively to the images of (2) only the C–H stretching region and to (1) all the 22 wavenumbers hyFOPM images (Extended Data Fig. 3a–c,e–g). In all three cases analyzed, linear unmixing separated Chol, DOPC and SM; however, not with the same accuracy. Crosstalk was characterized by tracing profiles along the wells of the unmixed images and plotting the mean intensity of the line profile as a pie chart. The SM was clearly distinguishable from other lipids in all the three cases (1), (2) and (3) (Fig. 1g and Extended Data Fig. 3d,h), Chol was well separated only in case (1) and (3) (Fig. 1g and Extended Data Fig. 3d,h). DOPC exhibited high crosstalk in all the three cases (Fig. 1g and Extended Data Fig. 3d,h). Overall, linear unmixing in the fingerprint region only (3) yielded the lowest crosstalk (Fig. 1g) compared with (1) or (2).

**Extended Data Fig. 4:**
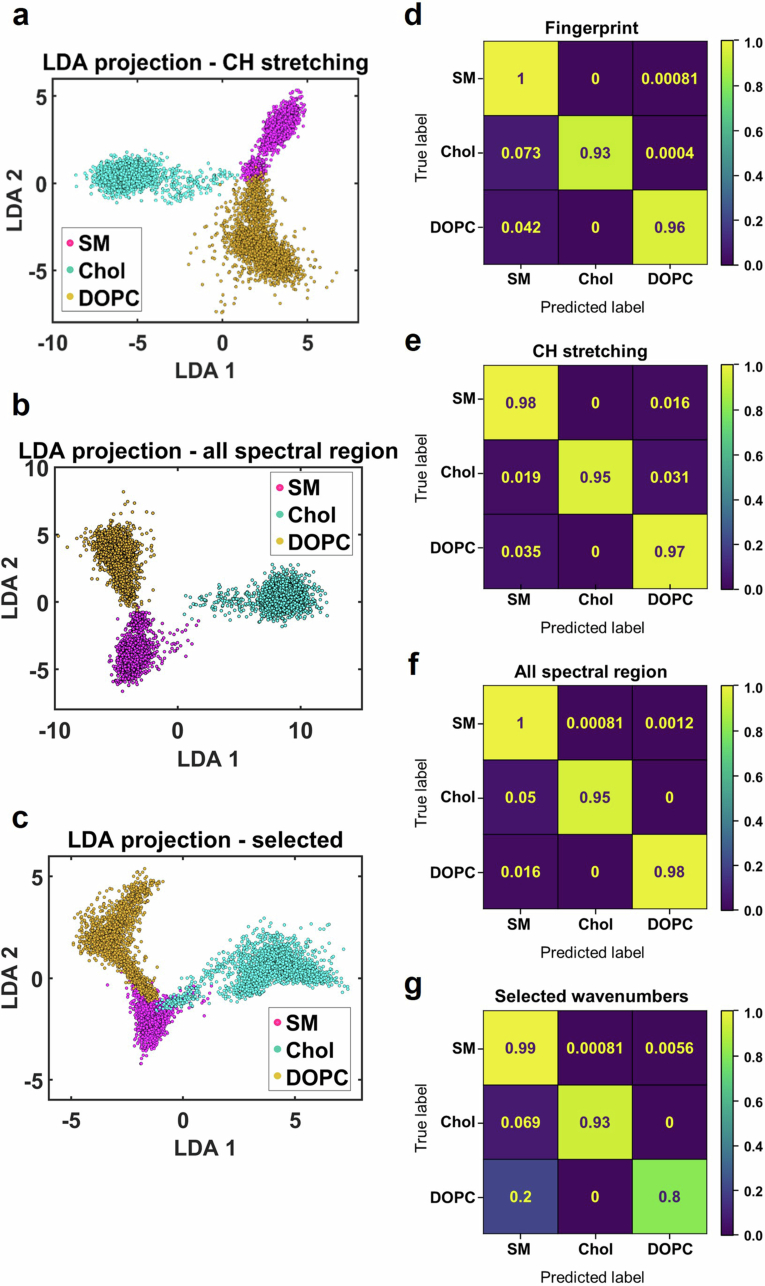
Linear Discriminant Analysis (LDA) and confusion matrixes. **a)** Scatter plot representing the LDA projections of hyperspectral fingerprint optoacoustic microscopy (hyFOPM) pixels obtained from images acquired in the CH stretching region. **b)** Scatter plot representing the LDA projections of hyFOPM pixels obtained from images acquired in the entire spectral region. **c)** Scatter plot representing the LDA of hyFOPM pixels obtained from images at selected wavenumbers (1731, 1645, 1555, 1464, 1375, 1056 cm^−1^). **(d-g)** Confusion matrixes extracted from the LDA analysis in Fig. 1g and (a-c). The confusion matrix in **(d)** originates from the LDA of the fingerprint pixels, the matrix in **(e)** from the LDA in the CH-stretching region, **(f)** from the LDA of all the spectral region, **(g)** from the LDA obtained for pixels at selected wavenumbers in the fingerprint region.

To improve spectral separation, we applied a linear discriminant analysis (LDA) to the three data sets (1, 2 and 3). LDA projection (Fig. 1h and Extended Data Fig. 4a,b) showed a clear class separation between the three lipids in all (1, 2 and 3) cases. The performance of the LDA classification was evaluated with confusion matrixes, which indicated an average comparable accuracy of 96% in classifying the lipids when the (3) fingerprint or only (2) the C–H stretching images were used (Extended Data Fig. 4d,e) and an average accuracy of 97% when using the entire dataset of images (Extended Data Fig. 4f). We also applied LDA to a subset of fingerprint wavenumbers (1,731, 1,645, 1,555, 1,464, 1,375 and 1,056 cm^−1^), selected by considering the strong absorption peaks of the three lipids in Fig. 1a–c. LDA projection of hyFOPM pixels acquired at selected wavenumbers showed distinguishable grouping between the three lipids (Extended Data Fig. 4c), with confusion matrixes revealed an accurate labeling for SM and Chol and a mislabeling of 20% for DOPC (Extended Data Fig. 4g), owing to the similarity with SM.

**Extended Data Fig. 5:**
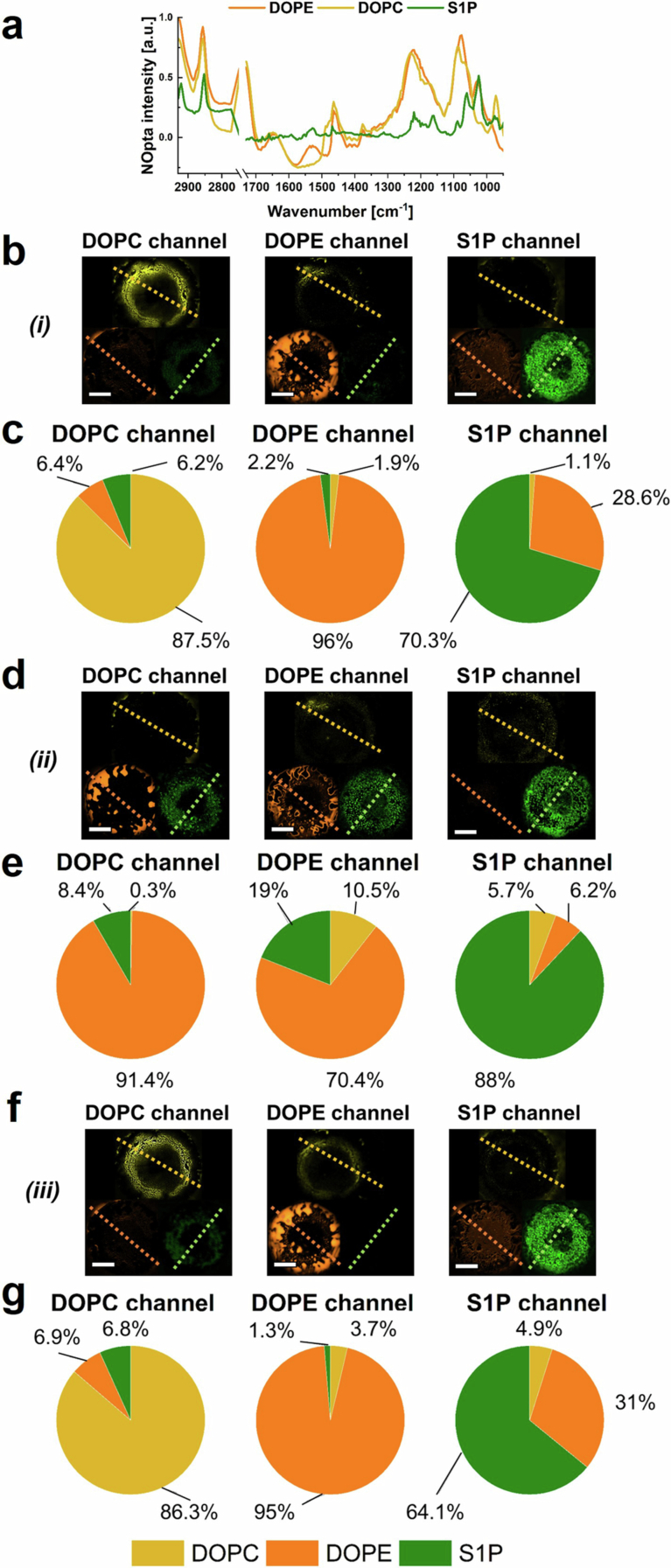
Linear unmixing and crosstalk pie charts for 2,3-dioleoyl-glycero-1-phosphocholine (DOPC), dioleoyl phosphatidylethanolamine (DOPE) and sphingosine-1-phosphate (S1P). **a)** Comparison of mid-infrared absorption spectra of DOPC, DOPE and S1P. **b)** Lipid maps from linear unmixing of hyperspectral fingerprint optoacoustic microscopy (hyFOPM) images acquired at thirty-seven wavenumbers covering the full spectral range *(i)*. **c)** Pie charts representing the crosstalk of the three lipid channels calculated from **(b)**. DOPC, DOPE, and S1P exhibited crosstalk levels of ~13%, 4%, and 29%, respectively. **d)** Lipids maps from linear unmixing of HyFOPM images acquired only in the C-H stretching spectral region *(ii)*. **e)** Pie charts representing the crosstalk of the three lipids channels calculated from **(d)**. DOPC, DOPE, and S1P exhibited crosstalk levels of ~99%, 30%, and 12%, respectively. **f)** Lipids maps from linear unmixing of hyFOPM images acquired only in the fingerprint spectral region *(iii)*. **g)** Pie charts representing the crosstalk of the three lipids channels calculated from **(f)**. DOPC, DOPE, and S1P exhibited crosstalk levels of ~14%, 5%, and 36%, respectively. Scale bar: 1 mm.

**Extended Data Fig. 6:**
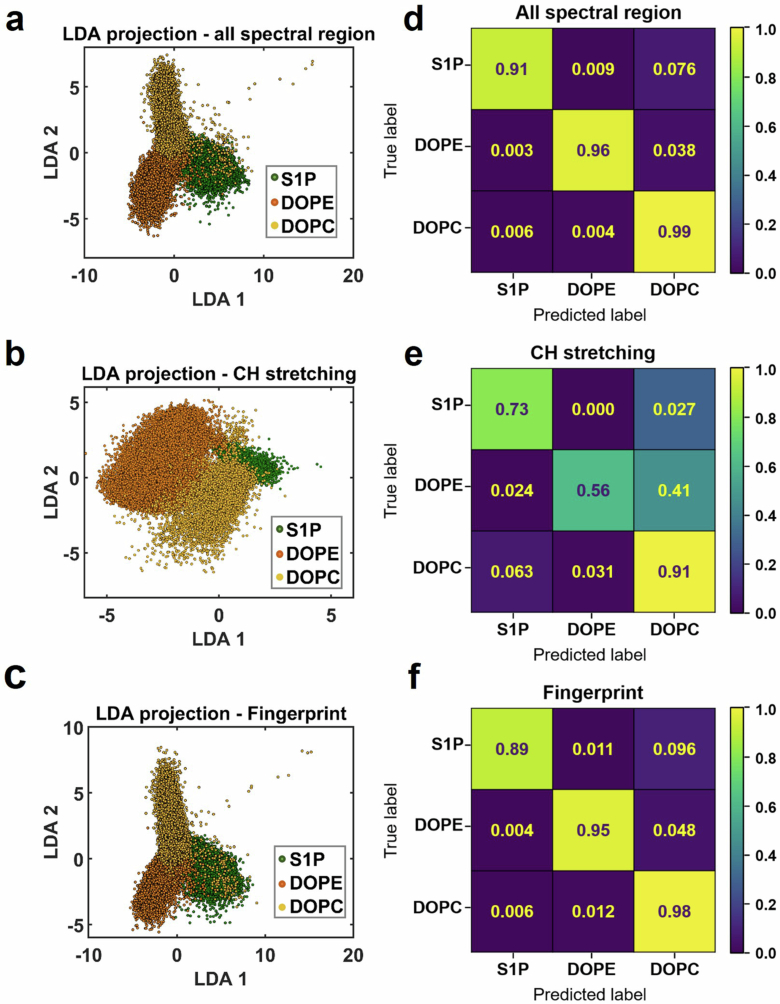
Linear discriminant analysis (LDA) and confusion matrixes. **(a-c)** Scatter plots representing LDA projections of hyperspectral fingerprint optoacoustic microscopy (hyFOPM) pixels obtained from images acquired at wavenumbers covering the **(a)** full spectral region, **(b)** only the C-H stretching region, and **(c)** only the fingerprint region. Confusion matrices originating from the LDA of **(d)** the full spectral region, **(e)** the C-H stretching region, **(f)** the fingerprint region.

To corroborate the hyFOPM ability to distinguish between lipids with similar vibrational modes and address potential cross-reactivity, we imaged a 2D phantom comprising DOPC, dioleoyl phosphatidylethanolamine (DOPE) and sphingosine-1-phosphate (S1P), which have similar absorption spectra (Extended Data Fig. 5a). The phantom was analyzed using linear unmixing (Extended Data Fig. 5b–g) and LDA (Extended Data Fig. 6) for the 1, 2 and 3 data-grouping cases.

### GUVs

**Fig. 2:**
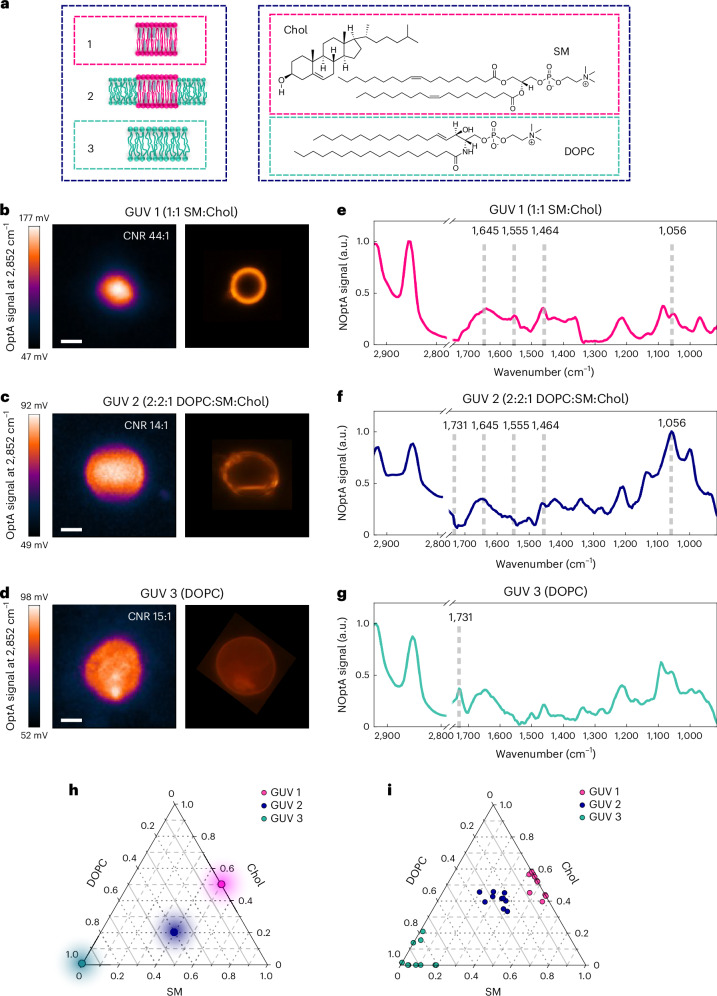
Lipid imaging and spectroscopy of GUVs by hyFOPM. **a**, Schematic models of GUVs with the corresponding lipid chemical structures drawn using ChemDraw Software. **b**–**d**, Left: hyFOPM images of GUV 1 (**b**), GUV 2 (**c**) and GUV 3 (**d**). Right: the corresponding fluorescent images. Scanning time for the acquisition of the hyFOPM images was 6 min. Scale bar, 25 μm, *N* = 3. **e**–**g**, HyFOPM spectra of GUVs in **b** to **d**. *N* = 75. **h**, Ternary phase diagram of DOPC [18:1(Δ9-Cis) PC], brain *N*-stearoyl-ᴅ-erythro-sphingosylphosphorylcholine [18:0 SM (d18:1/18:0)] and Chol >98% concentrations in GUVs. The vertices of the triangle represent pure components, while the edges are binary mixtures. The pink circular area corresponds to the molar ratio of GUV 1 composed by SM:Chol (1:1, molar ratio), the blue circular area corresponds to the molar ratio of GUV 2 composed by DOPC:SM:Chol (2:2:1, molar ratio), while the green circular area corresponds to GUV 3 composed by only DOPC. **i**, Ternary phase diagram obtained from linear unmixing of the spectra in **e** to **g**. The position of the dots corresponds to the contribution of the three different lipids. *N* = 10. OptA, optoacoustic. Source data

We used three types of GUVs at different Chol, SM and DOPC mixes to create biomimetic models of the plasma membrane (Fig. 2a). The ternary phase diagram (Fig. 2h) illustrates the assumed lipid formulation of the three GUV models examined, each represented by a circular area in the pyramid. The first model (Fig. 2b) contained a 1:1 mixture of SM and Chol, where the saturated acyl chains of SM and the rigid Chol molecules alternate forming a densely packed and ordered membrane (GUV 1). The second model (Fig. 2c) comprised DOPC, SM and Chol at a 2:2:1 molar ratio, a combination that creates tightly packed liquid ordered microdomains of SM and Chol, co-existing with a liquid disordered phase composed of DOPC (GUV 2). This type of membrane, where liquid ordered and liquid disordered phases coexist, simulates the plasma membrane of cells. Lastly, the third model (Fig. 2d) consists only of DOPC, which creates a purely fluid and disordered membrane (GUV 3).

HyFOPM images of the three GUVs, showing the C–H vibrations of lipid contrast at 2,852 cm^−1^, were corroborated by fluorescent images (Fig. 2b–d), using Nile red lipid staining. Although of lower resolution, due to the longer wavelengths used, hyFOPM images showed accurate anatomical correspondence with the fluorescent images. The GUV 1 contrast-to-noise ratio (CNR of 44; Fig. 2b) was ~3.1 times higher than the CNR of GUVs 2 and 3 (Fig. 2c,d, CNR of 14 and 15, respectively).

The higher CNR of GUV 1 may be explained by the higher local lipid concentration found in efficiently packed membranes compared with disordered ones. However, the presence of small SM:Chol domains did not greatly affect the local lipid concentration of GUV 2, which had a similar CNR of GUV 3.

**Extended Data Fig. 7:**
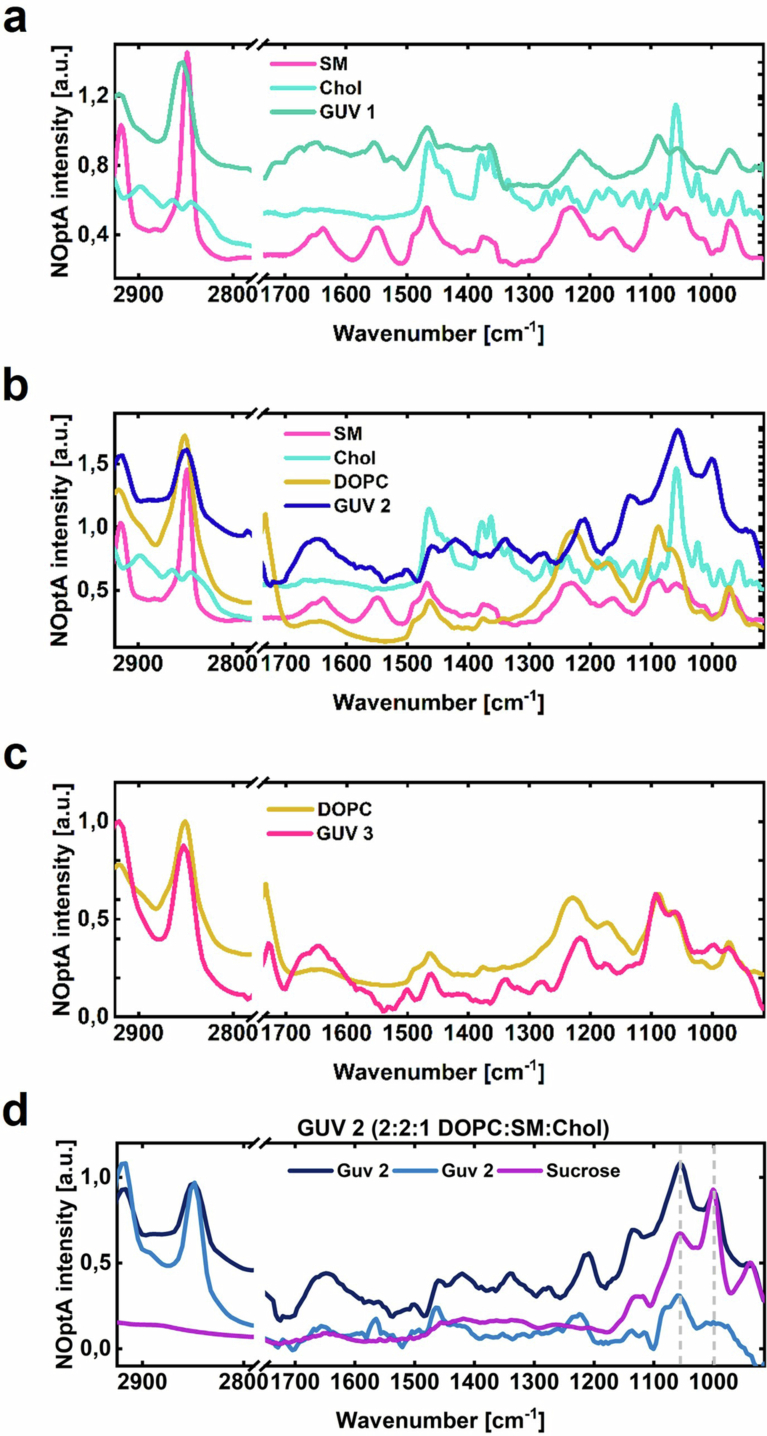
Comparison between spectra of Giant Unilamellar Vesicles (GUVs) and GUVs’ components. **(a)** Comparison between the spectra of cholesterol (Chol), sphingomyelin (SM) and the spectrum of GUV 1 (1:1, SM:Chol). **(b)** Comparison between the spectra of Chol, SM, phosphatidylcholine (DOPC), and the spectrum of GUV 2 (2:2:1 DOPC:SM:Chol). **(c)** Comparison between the spectrum of DOPC and the spectrum of GUV 3 (DOPC). **(d)** Spectra comparison of GUV 2 (dark blue) and sucrose (purple) used in GUV formation. The light blue line shows a GUV 2 example with minimal sucrose contribution. NOptA: Normalized Optoacoustic.

GUV spectra at the single-bond C–H stretching and the fingerprint regions were also acquired at different spatial locations on the GUV surface, and from different vesicles, with a spectral resolution of 4 cm^−1^ (Fig. 2e–g). The GUV 1 spectrum (Fig. 2e) unveiled contributions from the amide I–II and CH_2_ fatty acid chain SM peaks (at 1,645, 1,555 and 1,464 cm^−1^; Fig. 1c) and the sterol ring bending peak of Chol (at 1,056 cm^−1^; Fig. 1a). The GUV 1 spectrum is plotted next to the SM and Chol spectra (Extended Data Fig. 7a). The GUV 2 spectrum (Fig. 2f) exhibited peaks at 1,645, 1,555 and 1,464 cm^−1^, consistent with Fig. 1c, and a peak at 1,056 cm^−1^ representative of the Chol’s sterol ring (Fig. 1a). The DOPC presence gives rise to the shoulder detectable at 1,731 cm^−1^, as seen for DOPC (Fig. 1b). The spectral correlation between the GUV 2 spectrum and the spectra of the three lipids (Extended Data Fig. 7b) demonstrates that the peak at 1,000 cm^−1^ can be attributed to the presence of sucrose encapsulated inside the GUVs (Extended Data Fig. 7d). The DOPC-only GUV 3 (Fig. 2g) revealed an absorption spectrum similar to the one observed from the DOPC liquid solution (Fig. 1b and Extended Data Fig. 7c).

**Extended Data Fig. 8:**
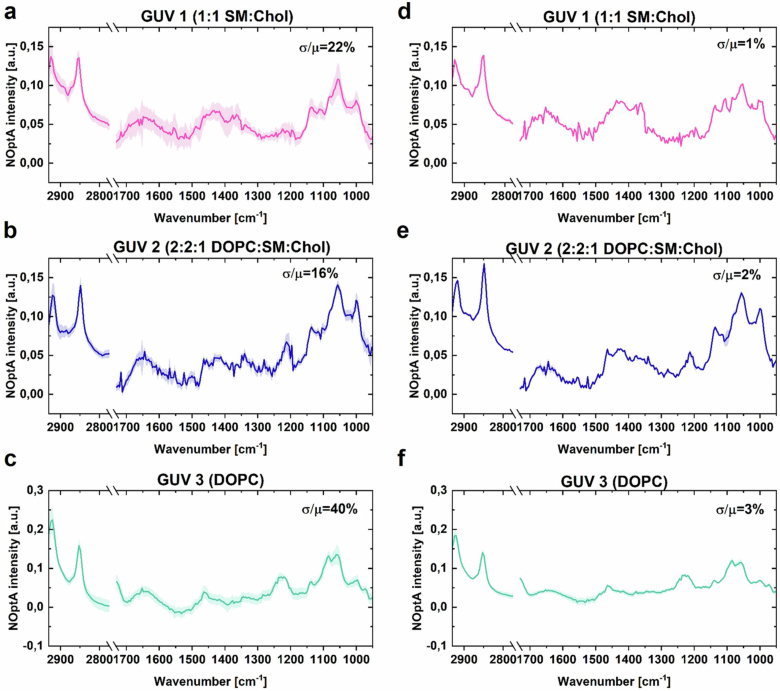
Giant unilamellar vesicles (GUV) spectra reproducibility. **a-c)** Mean (μ) and standard deviation (σ) of the ten spectral points collected for each GUV model reported in Fig. 2i. **a)** Mean and standard deviation of ten points in GUV 1 (1:1 SM:Chol) **b)** Mean and standard deviation of ten points in GUV 2 (2:2:1 DOPC:SM:Chol). **c)** Mean and standard deviation of ten points in GUV 3 (DOPC). **d)** Mean and standard deviation of two points located within the same GUV 1. **e)** Mean and standard deviation of three points located within the same GUV 2. **f)** Mean and standard deviation of five points located within the same GUV 3. The coefficient of variation is expressed as σ/ μ. DOPC: 1,2-dioleoyl-sn-glycero-3-phosphocholine. Chol: cholesterol. SM: sphingomyelin. NOptA: Normalized Optoacoustic.

Linear unmixing of the GUV spectra examined the spectral variability of the three GUV types and the intervariability within the same GUV type due to variations in GUV production. We performed spectral measurements on ten different vesicles from each GUV type (Extended Data Fig. 8a–c) that are rendered as a cloud of dots on the ternary plot of Fig. 2i and depict the actual relative concentration of each lipid component in the GUV, over the targeted concentration (Fig. 2h). The GUV 1 cloud (pink) lies in the middle of the edge between Chol and SM, indicating a binary mixture between these two endmembers. The GUV 2 cloud (blue) appears at the center of the plot, suggesting the presence of all three lipids, while the GUV 3 cloud (green) is located near the DOPC vertex. To validate that the observed variability is due to actual variations in GUV lipid composition, we performed spectra reproducibility measurements, at different spatial points within the same GUV (Extended Data Fig. 8d–f), which demonstrated a markedly lower variation than spectra acquired from different GUVs (Extended Data Fig. 8a–c). This finding points to hyFOPM as a potent tool for characterizing the actual lipid composition of GUVs, thus enhancing the accuracy of GUV research.

### Live cells

**Fig. 3:**
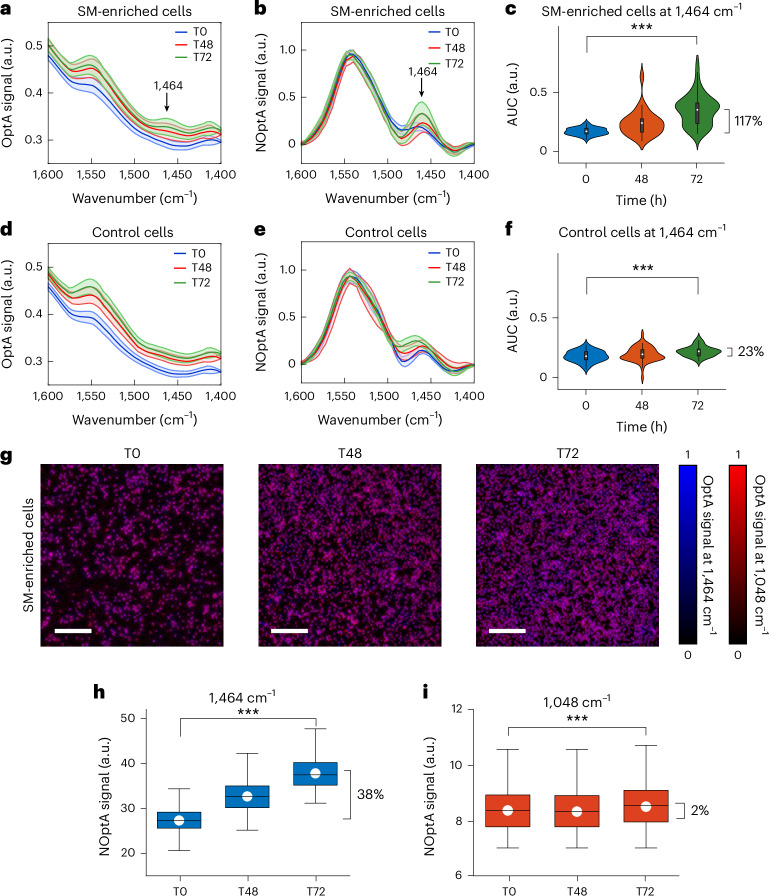
Imaging and spectroscopy of A549 SM-enriched cells upon 2-OHOA therapy. **a**,**d**, Raw spectra of SM-enriched A549 cells (**a**) and control (**d**) at different time points (T0, 0 h; T48, 48 h; T72, 72 h). **b**,**e**, Normalized and medium subtracted spectra of SM-enriched A549 cells (**b**) and control (**e**). Spectra were acquired in 50 cells. Data are presented as mean values ± s.d. **c**,**f**, Violin plots showing the kernel density of the AUC values for SM-enriched (**c**) and control (**f**) A549 cells at different time points. Box plot minima and maxima inside the violin plots indicate the interquartile range, the white circles indicate the mean values and the whiskers indicate the s.d. (coefficient of 1). Outliers are shown as individual points. ***P < 0.0001 (**c**) and *P* = 4.18 × 10^−13^ (**f**) from a two-sided paired *t*-test. *N* = 3. **g**, HyFOPM micrographs of SM-enriched cells at different time points (T0, 0 h; T48, 48 h T48; T72, 72 h). Overlay maps are obtained from SM (1,464 cm^−1^) and Chol contrast (1,048 cm^−1^). **h**,**i**, Box plots representing the OptA contrast of the micrographs in **g**: the SM contrast (1,464 cm^−1^) (**h**) and the Chol contrast (1,048 cm^−1^) (**i**) for 2-OHOA-stimulated A549 cells. Box plots indicate the standard error of the mean (s.e.m.; coefficient of 1; box limits), the white circles indicate the mean values, while the centered lines indicate the median values, and the whiskers indicate the s.d. (coefficient of 1). Outliers are shown as individual points. ***P < 0.0001 (**h**) and *P* = 9.59 × 10^−4^ (**i**) from a two-sided paired *t*-test. Scale bar, 300 μm, *N* = 3. OptA, optoacoustic; NOptA, normalized optoacoustic. Source data

We examined hyFOPM lipid detection in living cells, by resolving SM in 50 human lung adenocarcinoma cancer cells (A549), upon exposure to 2-hydroxyoleic acid (2-OHOA), an antitumor compound^32^. HyFOPM’s spectra were acquired with 2 cm^−1^ spectral resolution before and after cell treatment with 200 μM of 2-OHOA in the SM fingerprint region (1,600–1,400 cm^−1^) and revealed the expected increase in SM-related peaks (for example, 1,464 cm^−1^) after treatment (Fig. 3a,b). Violin plots exhibited an increase in the area under the curve (AUC) at 1,464 cm^−1^ by 117%, after 72 h of exposure to 2-OHOA and two cell distributions, that is, cells enriched in SM and not (Fig. 3c). For comparison, we also investigated the spectral content of 50 A549 cells maintained in culture for 72 h without 2-OHOA stimulation (Fig. 3d,e), demonstrating an AUC increase of only 23% (Fig. 3f).

**Extended Data Fig. 9:**
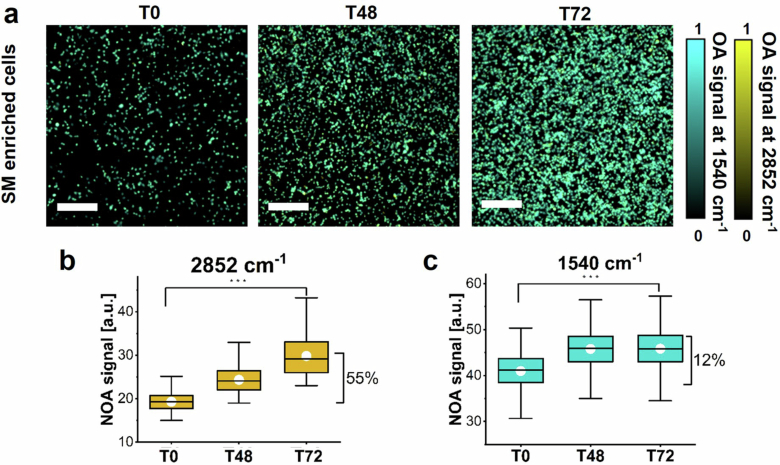
Spectral imaging of A549 sphingomyelin (SM) enriched cells upon 2-hydroxyoleic acid (2OHOA) treatment. **a)** Images monitoring at different time points (0 h = T0, 48 h = T48, and 72 h = T72) the accumulation of SM in A549 cells at 1540 cm^−1^ (protein contrast), and 2852 cm^−1^ (lipid contrast). Boxplots representing the optoacoustic (OptA) contrast of A549 cells in **(a)** at **(b)** 2852 cm^−1^ (lipid contrast) and at **(c)** 1540 cm^−1^ (protein contrast). Box plots indicate the standard error (SE, coefficient =1; box limits), the white circles indicate the mean values, while the centered lines the median values, and the whiskers indicate the standard deviation (SD, coefficient =1). ***p = 1.92 × 10^−241^ (b) and 1.21 × 10^−63^ (**c**) from a two-sided paired t-test. N = 3. NOptA: Normalized Optoacoustic.

With the 1,464 cm^−1^ peak confirmed for SM in phantoms, GUVs and living cells, we repeated the hyFOPM measurement in 3,000 A549 cells using only four selected wavenumbers: 2,852 cm^−1^ (total C–H lipid contrast), 1,540 cm^−1^ (amide II protein contrast), 1,464 cm^−1^ (SM contrast) and 1,048 cm^−1^ (Chol contrast), to examine selectivity with a small number of spectral measurements (Fig. 3g and Extended Data Fig. 9a). The measurements confirmed the previous observations, with 2-OHOA-stimulated A549 cells showing an increase in SM (blue) in Fig. 3g,h, observed at three different time points (time 0, 48 and 72 h after 2-OHOA exposure) but not in Chol (Fig. 3i, red). The SM increase as a function of time was also visible in the total lipid response (2,852 cm^−1^), whereby the protein contrast increased slightly (Extended Data Fig. 9b,c).

**Fig. 4:**
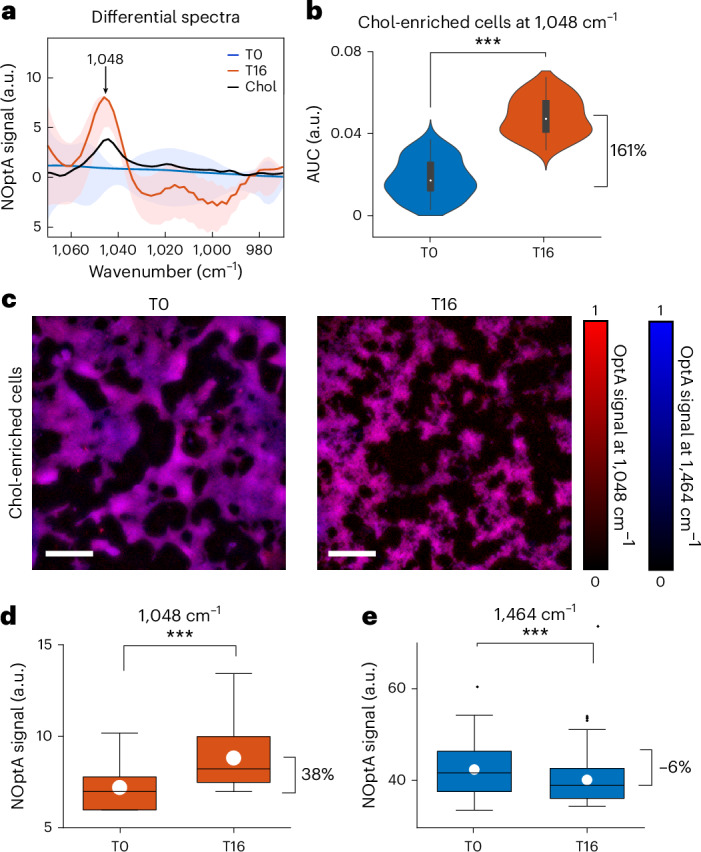
Imaging and spectroscopy of HEK cells after Chol loading via incubation with MβCD complexed with Chol. **a**, Differential spectra between 50 Chol-enriched and unenriched HEK cells. Data are presented as mean values ± s.d. For comparison, MβCD–Chol complex spectrum acquired with ATR–FTIR spectroscopy is shown in black. Chol-enriched HEK cells showed a peak at 1,048 cm^−1^, corresponding to the absorption peak of the MβCD–Chol complex. **b**, Violin plots showing the kernel density of the AUC of the peak at 1,048 cm^−1^ before (T0) and 16 h (T16) after exposure to the MβCD–Chol complex. Box plot minima and maxima inside the violin plots indicate the interquartile range, the white circles indicate the mean values and the whiskers indicate the s.d. (coefficient of 1). Outliers are shown as individual points. ***P = 1.46 × 10^−28^ from a two-sided paired *t*-test. *N* = 3. **c**, HyFOPM micrographs of HEK cells before (T0) and after treatment (T16) with MβCD–Chol complexes. Overlay maps are obtained from Chol (1,048 cm^−1^) and SM contrast (1,464 cm^−1^). **d**,**e**, Box plots representing the OptA contrast of the micrographs in **c**: the Chol contrast (1,048 cm^−1^) (**d**) and the SM contrast (1,464 cm^−1^) (**e**) for MβCD-treated HEK cells at time 0 h (T0) and 16 h (T16). Box plots indicate the s.e.m. (coefficient of 1; box limits), the white circles indicate the mean values, while the centered lines the median values, and the whiskers indicate the s.d. (coefficient of 1). Outliers are shown as individual points. ***P = 6.19 × 10^−12^ (**d**) and *P* = 5.48 × 10^−8^ (**e**) from a two-sided paired *t*-test. Scale bar, 300 μm, *N* = 3. OptA, optoacoustic; NOptA, normalized optoacoustic. Source data

**Extended Data Fig. 10:**
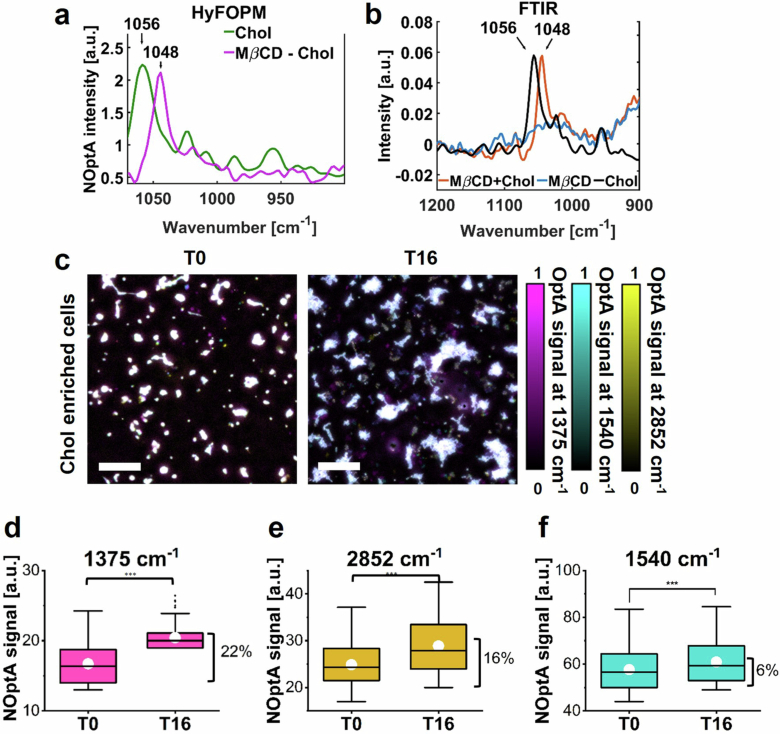
Spectral imaging of HEK cells after cholesterol (Chol) loading via incubation with methyl-β-cyclodextrins (MβCD) complexed with Chol. **a)** Comparison between the hyperspectral fingerprint optoacoustic microscopy (hyFOPM)’s spectra of a Chol liquid solution (green line) and of MβCD - Chol complex (purple line). The Chol absorption peak shifts from 1056 cm^−1^ to 1048 cm^−1^ when Chol is complexed with cyclodextrins. **b)** Comparison between the Fourier transform infrared (FTIR) spectra of MβCD complex with Chol (in red) and MβCD without Chol (in blue). For comparison, the FTIR spectrum of a Chol liquid solution is plotted in black. **c)** HyFOPM images monitoring Chol loading in HEK cells at 1375 cm^−1^ (Chol contrast), at 1540 cm^−1^ (protein contrast), and at 2852 cm^−1^ (lipid contrast) before (T0) and after 16 hours (T16) of treatment with MβCD-Chol. Boxplots representing the optoacoustic contrast of HEK cells in **(c)** at **(d)** 1375 cm^−1^ (Chol contrast), at **(e)** 2852 cm^−1^ (lipid contrast) and at **(f)** 1540 cm^−1^ (protein contrast). Box plots indicate the standard error (SE, coefficient =1; box limits), the white circles indicate the mean values, while the centered lines the median values, and the whiskers indicate the standard deviation (SD, coefficient =1). Outliers are shown as individual points. ***p = 2.24 × 10^−28^ (**d**), p = 3.16 × 10^−8^ (**e**) and p = 0.0018 (**f**) from a two-sided paired t-test. N = 3. OptA: Optoacoustic; NOptA: Normalized Optoacoustic.

In a second experiment, HEK cells (HEK293) were incubated with methyl-β-cyclodextrins (MβCD) complexed with Chol, to increase their membrane Chol content. MβCDs constituted a cavity that can carry hydrophobic molecules such as Chol; Chol is then delivered to the cell membrane^33^. HyFOPM’s spectra of 50 HEK cells before (T0) and after incubation (T16) demonstrated a prominent change at 1,048 cm^−1^ (Fig. 4a). There is an 8 cm^−1^ shift, between the 1,048 cm^−1^ and the 1,056 cm^−1^ peak where Chol was observed in the phantom (Fig. 1a) and in the GUV (Fig. 2e,f) measurement, due to the interaction of Chol with MβCD, resulting in an MβCD–Chol complex (Extended Data Fig. 10a,b). Violin plots of Chol demonstrated an AUC increase of 161% compared with the pre-exposure values (Fig. 4b), demonstrating a strong signal enhancement.

Chol detection from ~3,000 HEK cells was performed at five selected wavenumbers: 2,852 cm^−1^ (total C–H lipid contrast), 1,540 cm^−1^ (amide II protein contrast), 1,375 and 1,048 cm^−1^ (Chol contrast), and 1,464 cm^−1^ (SM contrast), confirming the Chol increase after 16 h incubation at 1,048 and 1,375 cm^−1^ (Fig. 4d and Extended Data Fig. 10d) and at the total lipid hyFOPM contrast at 2,852 cm^−1^ (Extended Data Fig. 10e). By contrast, the protein hyFOPM signal at 1,540 cm^−1^ (amide II) did not show a considerable increase after incubation (Extended Data Fig. 10f), and the 1,464 cm^−1^ wavenumber (SM contrast) showcased a slight decrease after incubation (Fig. 4e). This SM decrease can be explained by the known MβCD–Chol complex behavior to donate Chol while extracting other lipids from the plasma membrane^33,34^.

## Discussion

The ability to identify specific lipid classes in GUVs and in cells is essential to a broad range of applications, from understanding dynamic relationships in lipid metabolism to studying a variety of health conditions. Observation of SM and Chol, in particular, can enhance research abilities associated with a broad spectrum of diseases, owing the implication of these two lipids in atherosclerosis and stroke^35^, cognitive and neurodegenerative diseases^36^ or cancer^37^. We showed that the positive contrast of mid-IR optoacoustic detection and hyperspectral illumination enabled label-free detection of lipids in living cells, moving away from the use of fluorescence or isotope labels. We further found that the spectral specificity achieved in the fingerprint region (1,739–900 cm^−1^) was better than in the C–H stretching region (2,932–2,770 cm^−1^).

Label-free hyFOPM avoids the costly and lengthy development of specific labels and is not challenged by label delivery efficiency, selectivity and possible alteration of cellular function. By contrast, lipid selectivity is achieved by tuning the hyFOPM operation to the spectral signatures of the lipids of interest, which can be dynamically adapted to the requirements of the investigation performed. Detection characteristics in this case relate to the ability to differentiate the spectrum of the lipid of interest within the collected spectrum, which contains contributions from spectra of other background molecules. While established spectral unmixing techniques were used herein to demonstrate that sufficient information for SM and Chol differentiation is contained in the spectra collected, in a deterministic manner, advanced spectral unmixing techniques, including the use of deep learning, can help to improve the sensitivity and specificity limits of the technique. Besides observing lipids in living cells, hyFOPM can also be used to characterize the composition of GUVs, improving quality control in their production, especially given that we found variations up to 40% in the lipid composition of GUVs over the assumed ones.

Mid-IR optoacoustic microscopy can sample at >150-µm depths in tissues^19,38^. Therefore, a next step is to apply hyFOPM at thicker specimens or in vivo. Other future developments should be aimed at hyFOPM acceleration, through optimal illumination and spectral processing techniques that allow spectral subsampling, and miniaturization, so that the method can be applied to bedside analysis or disseminated laboratory use associated with diagnosing lipid disorders^39–42^.

## Methods

### HyFOPM description

The hyFOPM (Extended Data Fig. 1) experimental setup comprised a custom-built microscope configured for hyperspectral mid-IR excitation and OptA detection. A quantum cascade laser (QCL; Mircat, Daylight solutions) served as the mid-IR source, tunable between 3.4 and 11.0 μm (2,932–900 cm^−1^) with a spectral linewidth of ≤1 cm^−1^ (full-width at half maximum). The QCL emitted 20 ns pulses at a repetition rate of 100 kHz. The mid-IR beam was directed via a set of gold-coated mirrors, expanded using a beam expander and then focused onto the sample plane by a reflective objective with a numerical aperture of 0.5 (36×, Newport Corporation). The optical beam path was enclosed within a chamber continuously purged with N_2_ gas to eliminate interference from atmospheric CO_2_ and water vapor. Optical reference was provided by a mercury–cadmium–tellurium detector (Daylight Solutions), whose alignment with the QCL beam was facilitated with a visible laser pointer.

Samples were positioned on custom-made ZnSe (Edmund Optics) or ZnS (Crystal GmbH) dishes, which were placed on customized sample holders affixed to a motorized XYZ stage (Prior Scientific). Mid-IR absorption maps of samples were obtained using OptA signals detected by scanning a 25 MHz central frequency piezoelectric ultrasound transducer (Imasonic) immersed in coupling medium (deionized water or cell culture medium) along the focal plane. The motorized stage is capable of scanning up to a field of view (FOV) of 52 mm^2^ and a maximum velocity of 20 mm/s. The focused ultrasound transducer and the reflective objective are coaxially aligned to share the same focal plane as the sample. The raw OptA signals were amplified with a 63 dB low-noise amplifier (Narda-MITEQ) and filtered by a 50 MHz low pass filter (Mini-circuits). OptA signals were recovered using a data acquisition system (DAQ, Gage Applied) with a sampling rate of 200 MS/s. The hyFOPM system was controlled using custom software implemented in Matlab. HyFOPM has a spatial resolution of 4.3 μm and an axial resolution of 45.2 μm at 2,850 cm^−1^ that have been experimentally determined by measuring the point-spread-function of 1 μm polystyrene beads at 2,850 cm^−1^.

### CNR

The CNR was calculated as follows:

$$\mathrm{CNR}=\left|{\mathrm{OA}}_{{\rm{S}}}-{\mathrm{OA}}_{{\rm{B}}}\right|/{\mathrm{Noise}}_{\mathrm{PkPk}},$$

where OA_s_ and OA_B_ are the intensities of a point in the sample and one in the background, respectively, and Noise_PkPk_, is the peak-to-peak amplitude of the noise level.

### 2D lipid-solution phantom

DOPC [18:1(Δ9-Cis) PC], brain *N*-stearoyl-D-erythro-sphingosylphosphorylcholine [18:0 SM (d18:1/18:0)], Chol >98%, DOPE and S1P solutions (Avanti Polar Lipids) were dissolved in a solution of CHCl_3_:MeOH (2:1 v/v) and placed on wells using carbon tape as a spacer and reference. Micrographs of the 2D lipid phantom, covering a 10 mm × 10 mm FOV with a 25 μm step size, were acquired at selected wavenumbers every 25 min.

### Spectral unmixing and LDA

For spectral analysis of the 2D phantom, the hyFOPM images were preprocessed by normalizing the image intensity at each wavenumber by the mean intensity of the spectrally flat carbon tape, thus compensating for instability of the imaging system over time. Furthermore, each pixel of the hyFOPM image was normalized by its L1-norm along the wavenumber dimension. Spectral unmixing was then performed individually for each pixel of a hyFOPM image with a non-negative least squares solver, using pre-recorded hyFOPM spectra of the pure lipids, water and carbon tape as endmembers. The endmember spectra were normalized by their L1-norm. An LDA model was fit to manually labeled subsections of the three lipid wells shown in Fig. 1d–f with 50 × 50 pixels each. For the 2D projections shown in Fig. 1h and Extended Data Figs. 4 and 6, the LDA was given all pixels of the selected subsections for supervised dimensionality reduction. For the evaluation of the classification performance using different subsets of wavenumbers, shown as confusion matrixes in Extended Data Figs. 4 and 6, the LDA was trained and tested in a fivefold cross-validation scheme.

### PVA-assisted GUVs formation

The polyvinyl alcohol (PVA)-assisted method, as described in ref. ^43^, was adapted to prepare three types of GUVs with distinct lipid compositions, that is, (1) GUV 1, composed of SM and Chol in a 1:1 molar ratio; (2) GUV 2, containing DOPC, SM and Chol in a 2:2:1 molar ratio; and (3) GUV 3, consisting only of DOPC. All three lipid stock solutions were first prepared at a concentration of 1 mg/ml in a ratio of 1:1 CHCl_3_:MeOH and then mixed in the specified ratios to form the three lipid compositions. Each lipid solution was added to a PVA-coated vial and heated to 50 °C to remove the solvents. The PVA coating was created by dissolving PVA fully hydrolyzed powder (Sigma-Aldrich) in 280 mM sucrose (Sigma-Aldrich) and heating it to 90 °C until completely dissolved, resulting into a 5% (w/v) PVA solution. A 200 µl aliquot of the PVA solution was then transferred into a 5 ml glass vial and placed in a desiccator overnight, allowing the solution to vaporize and to create a uniform thin PVA coating. To induce GUV formation, 280 mM sucrose was added to the lipid–PVA mixture, followed by incubation at 37 °C for 20 min. Afterward, 300 µL additional sucrose was added to suspend the GUVs. A 200 µl aliquot of the GUV suspension was, finally, sedimented on the imaging dish using phosphate-buffered saline. The scanning time required for hyFOPM images of GUVs was 6 min.

### Cell culture

Accumulation of SM and Chol was performed respectively on a human adenocarcinoma alveolar basal epithelial cell line (A549, American Type Culture Collection (ATCC): CCL-185) and a HEK cell line (HEK293, ATCC: CRL-1573). A549 cells were cultured in Dulbecco’s Modified Eagle Medium/Nutrient Mixture F12 (DMEM/F12, Gibco) supplemented with 10% fetal bovine serum (FBS, Merck,) and 1% penicillin–streptomycin (Sigma-Aldrich). HEK293 cells were cultured in Minimum Essential Medium (MEM, Gibco) supplemented with 10% FBS and 1% penicillin–streptomycin. Cells were grown in an incubator at 37 °C, 5% CO_2_ and 20% O_2_. Accumulation of SM was induced via treatment with 200 μM 2-OHOA solution (Avanti Polar Lipids). Spectra and images of A549 treated cells were recorded at time 0 h, time 48 h and time 72 h (T0, T48 and T72). Accumulation of Chol was induced via treatment with 1 mM MβCD–Chol complex (Sigma-Aldrich). Spectra and images of HEK treated cells were recorded after 16 h of exposure to MβCD–Chol complex.

### Analysis of spectra

For statistical elaboration, 50 spectra of A549 and HEK cells were acquired in the fingerprint skeletal range (from 1,600 to 1,400 cm^−1^ for A549 cells, and from 1,070 to 900 cm^−1^ for HEK cells). In both live cell experiments, spectra were preprocessed by removing the baseline offset by subtraction of the first spectral value, followed by normalization to the spectrum’s final point. The corrected spectra were then interpolated along the spectral range using a spline function to ensure consistent sampling for all measurements. To minimize background or medium contribution, the corresponding background or medium-normalized spectrum was subtracted from the spectrum of the cells. Finally, spectra were normalized to their maximum intensity to facilitate comparison between samples. In the case of Chol accumulation, differential spectra between normalized spectra acquired at time 0 (T0) and spectra at time 16 h (T16) were calculated. The spectra shown in Figs. 3b,e and 4a correspond to mean values ± standard deviation (s.d.) values obtained from 50 different cells. Violin plots (Figs. 3c,f and 4b) were generated on the basis of AUC values for the band spanning 1,484–1,424 cm^−1^ (Fig. 3b,e) and the band spanning 1,062–1,026 cm^−1^ (Fig. 4a). Kernel density estimation was applied to visualize the data distribution. Each plot shows the probability density of the dataset, mirrored vertically, with embedded box plot elements (median and interquartile range) included. Matlab 2019 and OriginPro9 software were used for data analysis.

### Image processing

HyFOPM micrographs of A549 and HEK living cells with an FOV of 1.5 mm × 1.5 mm (pixel size of 5 μm, 50 average) were acquired at different wavenumbers (2,852, 1,540, 1,464, 1,375 and 1,048 cm^−1^), as shown in Figs. 3g and 4c and Extended Data Figs. 9a and 10c. The scanning time required for each micrograph was 15 min per wavenumber. The hyFOPM micrographs were analyzed using ImageJ software. First, micrographs were normalized by background/medium subtraction, and then the region of interest containing cells was segmented and the OptA intensity of the cells was extracted. The optoacoustic intensity extracted from the micrographs is shown in the form of box plots in Figs. 3h,i and 4d,e and Extended Data Figs. 9b,c and 10d–f.

### Reporting summary

Further information on research design is available in the Nature Portfolio Reporting Summary linked to this article.

## Online content

Any methods, additional references, Nature Portfolio reporting summaries, source data, extended data, supplementary information, acknowledgements, peer review information; details of author contributions and competing interests; and statements of data and code availability are available at 10.1038/s41592-026-03025-w.

## Supplementary information

Reporting Summary

Supplementary Video 1 Spectral images of the lipid phantom acquired by hyperspectral fingerprint optoacoustic microscopy (hyFOPM).

## Source data

Source Data Fig. 1 Numerical source data.

Source Data Fig. 2 Numerical source data.

Source Data Fig. 3 Numerical source data.

Source Data Fig. 4 Numerical source data.

## Data Availability

The data supporting the findings of this study are available upon reasonable request from the corresponding author because they are currently in use for other publications. Source data are provided with this paper.
